# Editorial: Materials, adaptation, and evolution: how anthropogenic activities impact microbial resistance

**DOI:** 10.3389/fmicb.2026.1960288

**Published:** 2026-08-21

**Authors:** Paolo Pellegrino, Fabrizio Damiano, Chiara Leo, Matteo Calcagnile

**Affiliations:** 1 Department of Mathematics and Physics “E. De Giorgi”, University of Salento, Lecce, Italy; 2 Institute for Microelectronics and Microsystems (IMM), CNR, Lecce, Italy; 3 Department of Experimental Medicine (DiMeS), University of Salento, Lecce, Italy; 4 NGS and Bioinformatic Laboratory, Polo d'Innovazione di Genomica, Genetica e Biologia SRL, Terni, Italy; 5 Department of Biological and Environmental Sciences and Technologies (DiSTeBA), University of Salento, Lecce, Italy

**Keywords:** anthropogenic materials, antimicrobial materials, antimicrobial resistance, microbial evolution, nanomaterials

Antimicrobial resistance (AMR) is one of the defining challenges of modern medicine, threatening decades of progress in the treatment of infectious diseases. While the misuse and overuse of antibiotics remain the principal reason for AMR, there is growing recognition that microbial evolution is influenced by a much broader range of anthropogenic pressures. Industrial pollution, urbanization, climate change, and the widespread dissemination of engineered materials have transformed microbial habitats, exposing microorganisms to environmental conditions that differ profoundly from those under which they evolved. As a result, microorganisms continuously adapt to novel physical, chemical, and biological stresses, often developing survival strategies that extend beyond classical antibiotic resistance.

Advanced materials play an important role in anthropogenic activities. Among these, nanomaterials, antimicrobial coatings, biomaterials, and functional polymers are increasingly used in healthcare, agriculture, food production, and environmental technologies. These new materials can inhibit microbial colonization, thereby reducing infections. However, these materials also create new ecological environments where microorganisms are constantly exposed to selective pressures that can change their physiology, behavior, and evolution. These interactions can influence biofilm formation, stress adaptation, horizontal gene transfer, and, ultimately, the emergence of resistant phenotypes. A deeper understanding of the interplay between materials and microorganisms is thus required for developing antimicrobial technologies that remain effective over time while minimizing unintended evolutionary consequences.

In this framework, the Research Topic titled *Materials, Adaptation, and Evolution: How Anthropogenic Activities Impact Microbial Resistance* has been conceived to foster an interdisciplinary dialogue between microbiologists, materials scientists, chemists, environmental researchers, and biomedical engineers. Rather than evaluating antimicrobial materials solely based on their ability to eliminate microorganisms, this Research Topic adopts a broader perspective, recognizing that materials actively interact with microbial communities and can both influence and respond to microbial adaptation.

Against this background, the five contributions included in this Research Topic provide complementary perspectives on the multifaceted relationship between anthropogenic materials and microbial adaptation. Collectively, they explore how environmental pressures influence microbial evolution while highlighting innovative material-based approaches to prevent and combat antimicrobial resistance.

The Research Topic begins with Morkoç et al.'s review, which provides a comprehensive overview of how anthropogenic pollutants, including heavy metals, organic contaminants, microplastics, and engineered nanomaterials, act as environmental stressors that influence microbial adaptation and resistance. The authors examined the responses of bacteria, fungi, and microalgae, highlighting the molecular mechanisms that underlieg microbial resilience and discussing how emerging multi-omics approaches are advancing our understanding of pollutant-specific adaptive strategies.

On the other hand, Sun et al. focused on the genetic mechanisms underlying antimicrobial resistance in *Mycobacterium tuberculosis*. Using a pan-genomic approach, the authors identified evolutionary signatures associated with drug resistance and proposed a drug-resistant mutation pattern that may support more informed drug selection and individualized therapeutic strategies.

Building on this mechanistic understanding, three original research articles explore how advanced materials can be engineered to prevent microbial infections using complementary strategies.

Abdallah et al. provide an elegant example of green nanotechnology for treating osteomyelitis. The authors report the phyto-fabrication of gold nanoparticles using the extract from *Desmidorchis retrospiciens*, demonstrating potent antibacterial activity against methicillin-resistant *Staphylococcus aureus* (MRSA) through ROS-mediated membrane damage and inhibition of biofilm formation. In addition to their antimicrobial efficacy, these biogenic nanoparticles also promote the osteogenic differentiation of bone marrow-derived mesenchymal stem cells and enhance bone regeneration without any detectable cytotoxicity.

A complementary strategy is presented by Tai et al. who developed hyaluronic acid-modified PLGA nanospheres for the controlled delivery of niclosamide against *Clostridioides difficile*. The nanocarrier improved the drug's solubility, stability, and pH-responsive release, significantly enhancing its therapeutic performance both *in vitro* and *in vivo*. In addition to preventing bacterial growth, the formulation significantly suppressed spore germination, biofilm formation, and toxin production in an animal model, resulting in reduced disease severity.

Zheng et al. complete the Research Topic by reporting the development of a photoacid-based crystalline coordination polymer with intrinsic antibacterial activity. This study demonstrates how the rational design of functional materials can result in innovative antimicrobial platforms with distinct physicochemical properties, expanding the toolbox available for combating pathogenic microorganisms.

Although the studies presented in this Research Topic differ in their objectives, methodologies, and experimental models, they share a common message: materials play an active role in shaping microbial behavior and evolution. Whether they are encountered as environmental pollutants, engineered nanomaterials, drug delivery systems, or functional biomaterials, materials continuously interact with microorganisms, influencing their physiology, adaptation, and survival. At the same time, advances in materials science are creating exciting opportunities to develop innovative strategies for preventing and treating microbial infections, as well as addressing the growing challenge of antimicrobial resistance.

Together, these contributions encourage a deeper understanding of how antimicrobial materials should be created and evaluated. Their success should be judged not only by their ability to inhibit microbial growth or eradicate pathogens but also by their long-term impact on microbial adaptation. Understanding how materials affect processes such as biofilm formation, stress responses, horizontal gene transfer, and the emergence of tolerant or resistant phenotypes will be essential for developing effective and sustainable antimicrobial technologies.

Finally, addressing these challenges requires an interdisciplinary approach that combines expertise from microbiology, materials science, environmental science, chemistry, evolutionary biology, and computational biology. Only by integrating these complementary disciplines can we fully understand the complex interactions between anthropogenic materials and microbial communities and then transfer this knowledge into safer and more effective antimicrobial solutions.

